# C-Reactive Protein Levels Predict Responses to PD-1 Inhibitors in Hepatocellular Carcinoma Patients

**DOI:** 10.3389/fimmu.2022.808101

**Published:** 2022-02-04

**Authors:** Yiyang Zhang, Lianghe Lu, Zhangping He, Zhishen Xu, Zhicheng Xiang, Run-Cong Nie, Wenping Lin, Wenxu Chen, Jie Zhou, Yixin Yin, Juanjuan Xie, Youcheng Zhang, Xueyi Zheng, Tianchen Zhu, Xiaoxia Cai, Peng Li, Xue Chao, Mu-Yan Cai

**Affiliations:** ^1^ Collaborative Innovation Center for Cancer Medicine, State Key Laboratory of Oncology in South China, Sun Yat-sen University Cancer Center, Guangzhou, China; ^2^ Department of Endoscopy, Sun Yat-sen University Cancer Center, Guangzhou, China; ^3^ Department of Pathology, Sun Yat-sen University Cancer Center, Guangzhou, China; ^4^ Department of Hepatobiliary Oncology, Sun Yat-sen University Cancer Center, Guangzhou, China; ^5^ Department of Gastric Surgery, Sun Yat-sen University Cancer Center, Guangzhou, China

**Keywords:** C-reactive protein, hepatocellular carcinoma, PD-1 inhibitors, prognosis, tumor response

## Abstract

**Background:**

Serum C-reactive protein (CRP) is a biomarker of an acute inflammatory response and has been successfully used as a prognostic predictor for several malignancies. However, the clinicopathological significance of CRP levels in hepatocellular carcinoma (HCC) patients being treated with PD-1 inhibitors remains unclear.

**Methods:**

Serum CRP levels were measured for a total of 101 HCC patients that had been treated with PD-1 inhibitors from July 2018 to November 2019. The clinicopathological data was retrospectively analyzed to identify any clinical implications between CRP levels and responses to PD-1 inhibitors and patients’ progression-free survival (PFS).

**Results:**

The median PFS was 8.87 months in the CRP-low subgroup and 3.67 months in the CRP-high subgroup (*P =* 0.009). Univariate and multivariate Cox regression analysis demonstrated that both serum CRP and AFP levels were independent risk factors for the PFS of HCC patients treated with PD-1 inhibitors (*P* < 0.05). Moreover, Cox regression analysis after Propensity Score Matching showed the similar results. A prognostic model combining CRP and AFP levels could significantly stratify HCC patients receiving PD-1 inhibitors into low-, intermediate-, and high-risk subgroups (*P* < 0.001). Patients in the risk subgroups reported similar overall response rates (*P =* 0.625) and significantly different disease control rates (low- vs. intermediate- vs. high-risk groups: 81.6% vs. 65.1% vs. 35%, respectively, *P =* 0.002).

**Conclusions:**

The results of this study support the association between high serum CRP levels with the response and PFS for HCC patients receiving PD-1 inhibitors. Furthermore, the levels of both CRP and AFP in an HCC patient before treatment initiation show great potential for determining the efficacy of PD-1 inhibitors.

## Introduction

Primary liver cancer is the sixth most common malignancy and the third leading cause of cancer deaths worldwide in 2020 (1). Hepatocellular carcinoma (HCC) comprised 75%-85% of the reported primary liver cancer cases. As a result of the strong and broad resistance of HCC to cytotoxic chemotherapy, systemic therapy has become a common alternative option. Oral multi-tyrosine kinase inhibitors (TKI) were shown to prolong the survival of patients with advanced HCC whose liver function was preserved (2, 3). However, there was no evidence of efficacy for TKIs in the treatment of advanced stage HCC when combined with transarterial chemoembolization (TACE) in the intermediate stage or when given to prevent recurrence after liver resection or ablation (4).

The immune system plays an essential role in suppressing cancer progression (5). Immunotherapies targeting the programmed cell death 1 (PD-1) receptor protein blockade has resulted in increased survival for some patients in many clinical trials encompassing a variety of cancer types (6, 7). However, only 20% to 40% of patients responded to these treatments and even fewer had long-term disease remission (8–10). The response to PD-1 inhibitors in HCC patients is remarkably heterogeneous, and our current understanding of which patients will benefit from PD-1 inhibitors is very limited. Thus, identifying a means of predicting the response of an HCC patient to PD-1 inhibitors is urgently needed.

Clinical research on checkpoint inhibitors had previously found that elevated CRP levels negatively correlated with clinical outcomes for melanoma when treated with checkpoint inhibitors (11, 12). Additionally, AFP produced by HCC has been shown to have a suppressive effect on NK cells and T cells, and DCs that have been exposed to AFP have been shown to have a reduced effect on the stimulation of antigen specific T cell activation and proliferation (13). Thus, we performed a retrospective study to determine whether pre-treatment serum CRP in combination with AFP levels could discriminate which HCC patients would likely benefit from receiving PD-1 inhibitors.

## Method and Materials

### Patients

The medical records of patients with histologically confirmed HCC who were treated with PD-1 inhibitors from July 2018 to November 2019 at the Sun Yat-sen University Cancer Center were summarized. The following data was then retrospectively reviewed for each patient: medical history, laboratory results, radiological results, and prior treatments before receiving PD-1 inhibitors. All follow-ups were from the initiation of PD-1 inhibitors until 30 March 2020. PD-1 inhibitors were all administered according to the following regimens: Nibolumab at 1-3 mg/kg body weight per 2 weeks, Pembrolizumab at 200mg per 3 weeks, Toripalimab and Sintilimab at 240 mg and 200 mg per 2 weeks. Demographic and clinical characteristics were summarized in **Table 1**. Radiological response was evaluated by computed tomography (CT) or magnetic resonance imaging (MRI) performed at a baseline, 6-12 weeks after treatment initiation, and approximately every 3 months thereafter according to the Response Evaluation Criteria in Solid Tumors (RECIST) v1.1.

**Table 1:** Baseline characteristics of HCC patients.

	Total (n = 101)	CRP > 20.9 (n = 28)	CRP ≤ 20.9 (n = 73)	*P value*
**Age (year)**				0.653
≤ 60	80 (79.2)	23 (82.1)	57 (78.1)	
> 60	21 (20.8)	5 (17.9)	16 (21.9)	
**Gender**				
Male	82 (81.2)	25 (89.3)	57 (78.1)	0.197
Female	19 (18.8)	3 (10.7)	16 (21.9)	
**Hepatitis B infection**				0.361
Yes	91 (90.1)	24 (85.7)	67 (91.8)	
No	10 (9.9)	4 (14.3)	6 (8.2)	
**Child-Pugh grade**				0.301
A	90 (89.1)	23 (82.1)	67 (91.8)	
B	11 (10.9)	5 (17.9)	6 (8.2)	
**AFP level (ng/l)**				0.034
≤ 400	46 (45.5)	8 (28.6)	38 (52.1)	
> 400	55 (54.5)	20 (71.4)	35 (47.9)	
**WBC count (10^9/L)**				0.025
<10	94 (93.1)	23 (82.1)	71 (97.3)	
>10	7 (6.9)	5 (17.9)	2 (2.7)	
**Neutrophil-lymphocyte ratio (NLR)**				0.001
<3	58 (57.4)	8 (28.6)	50 (68.5)	
≥3	43 (42.6)	20 (71.4)	23 (31.5)	
**ECOG PS**				0.082
0	39 (38.6)	7 (25.0)	32 (43.8)	
≥ 1	62 (61.4)	21 (75.0)	41 (56.2)	
**Tumor size (cm)**				0.001
≤ 5	49 (48.5)	6 (21.4)	43 (58.9)	
> 5	52 (51.5)	22 (78.6)	30 (41.1)	
**Tumor number**				0.408
≤ 1	39 (38.6)	9 (32.1)	30 (41.1)	
> 1	62 (61.4)	19 (67.9)	43 (58.9)	
**Macroscopic vascular invasion**				0.129
Present	49 (48.5)	17 (60.7)	32 (43.8)	
Absent	52 (51.5)	11 (39.3)	41 (56.2)	
**Extrahepatic metastasis**				0.459
Present	59 (58.4)	18 (64.3)	41 (56.2)	
Absent	42 (41.6)	10 (35.7)	32 (43.8)	
**BCLC stage**				0.302
A	7 (6.9)	3 (10.7)	4 (5.5)	
B	10 (9.9)	1 (3.6)	9 (12.3)	
C	84 (83.2)	24 (85.7)	60 (82.2)	
**Prior treatment**				0.651
Curative treatment (Surgery, Ablation)	41 (40.6)	7 (16.7)	34 (22.7)	
Local-regional (TACE, HAIC, radiation)	82 (81.2)	20 (47.6)	62 (41.3)	
Target therapy (Sorafenib, lenvatinib)	69 (68.3)	15 (35.7)	54 (36.0)	

Variables are expressed as n (%).

AFP, α-fetoprotein; BCLC stage, Barcelona Clinic Liver Cancer stage; CRP, C-reactive protein; ECOG PS, Eastern Cooperative Oncology Group performance status; HAIC, hepatic arterial infusion of chemotherapy; HCC, hepatocellular carcinoma; NLR, Neutrophil-lymphocyte ratio; TACE, transcatheter arterial chemoembolization.

### Data Collection

The demographic and clinical characteristics included age, gender, HBV infection, Child-Pugh grade, CRP levels (mg/L), AFP levels (ng/L), WBC count (10^9^/L), neutrophil-lymphocyte ratio (NLR), ECOG PS, tumor size (cm), tumor number, macroscopic vascular invasion, extrahepatic metastasis, BCLC stage, and any prior treatments.

The serum CRP and AFP levels were measured within a period of 5 days prior to the onset of receiving PD-1 inhibitors. All the reagents used for the detection of CRP and AFP met the WHO standards. Child-Pugh grade was collected based on variables including ascites, encephalopathy, serum albumin, bilirubin, and prothrombin time. Imaging tools including CT, MRI and/or ultrasonography were used to detect tumor size, number, macroscopic vascular invasion, lymph nodes and extrahepatic metastasis, and tumor recurrence. PFS was defined as the time from the initiation of receiving PD-1 inhibitors until HCC relapse, disease progression, or patient death from HCC. Tumor responses to PD-1 inhibitors included complete response (CR), partial response (PR), stable disease (SD), and progressive disease (PD) according to RECIST v1.1.

The methods used in this study were approved by the Institute Research Medical Ethics Committee of the Sun Yat-sen University Cancer Center.

### Statistical Analysis

**Figure 1:**
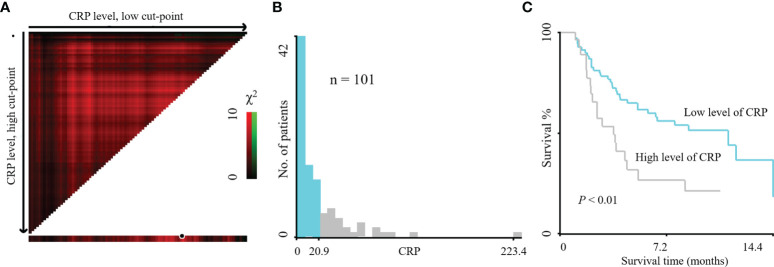
X-tile plots of the prognostic marker CRP levels on HCC patients treated with PD-1 inhibitors. X-tile plot showed the χ2 log-rank values created when the cohort was divided into two populations. The cut-point highlighted by the black/white circle **(A)** was demonstrated on a histogram of the entire cohort **(B)** and a Kaplan–Meier plot **(C)**. CRP levels were divided at the optimal cut-point, as defined by the most significant point on the plot (≤ 20.9 mg/L and > 20.9 mg/L of HCC patients treated with PD-1 inhibitors, *P < *0.01).

Categorical variables were analyzed using the Pearson chi-square test. The cutoff value for CRP (best cutoff value = 20.9 mg/L) was determined by X-tile application (**Figure 1**) (14). Independent predictive variables were determined with univariate and multivariate Cox regression analysis for PFS by the method of Forward LR. The risk stratification survival curves were represented by Kaplan-Meier curves and analyzed using the Log-rank test. CRP, AFP, and several other variables were analyzed using ROC curve analysis to evaluate overall response (ORR = CR + PR) and tumor progression of patients to PD-1 inhibitors. Statistical analyses were conducted using the IBM SPSS statistics version 19.0 (IBM, Armonk, NY, USA). Propensity Score Matching was conducted using the IBM SPSS statistics version 26 (IBM, Armonk, NY, USA) (15). Nomogram for predicting the PFS of HCC patients with CRP and AFP was conducted using R version 3.5.1.

## Results

### Patient Characteristics

A total of 101 patients with advanced HCC were treated with PD-1 inhibitors at the Sun Yat-sen University Cancer Center during the study period. The HCC cohort included 82 (81.2%) males and 19 (18.8%) females, with an average age of 49.4 years. The average follow-up time after immunotherapy was 7.7 months. The median duration of treatment was 2.8 months (95% confidence interval [CI]: 2.4 - 3.1 months). The median PFS time was 5.5 months (95% CI: 4.8 - 6.3 months). There were no significant differences in the demographics and clinical characteristics between the CRP-high and -low groups with the exception of AFP levels > 400 ng/L (CRP-high vs. -low groups = 71.4% vs. 47.9%, *P* = 0.034), WBC count >10×10^9^/L (17.9% vs. 2.7%, *P =* 0.025), NLR ≥ 3 (71.4% vs. 31.5%, *P* = 0.001), and tumor size > 5 cm (78.6% vs. 41.1%, *P* = 0.001). Prior to treatment with PD-1 inhibitors, a total of 78 (77.2%) patients had received other treatment regimens, including curative treatments, locoregional treatments (TACE, hepatic artery infusion chemotherapy (HAIC), and radiation) and targeted therapies (Sorafenib or Lenvatinib), which had failed or the patients were shown to be resistant. Twenty-three (22.8%) patients underwent neoadjuvant therapy, including 17 (73.9%) patients that had been treated with PD-1 inhibitors combined with HAIC and 6 (26.1%) with PD-1 inhibitors plus targeted therapy and HAIC (summarized in **Table 1**).

### Risk Factors for PFS of HCC Patients

**Figure 2:**
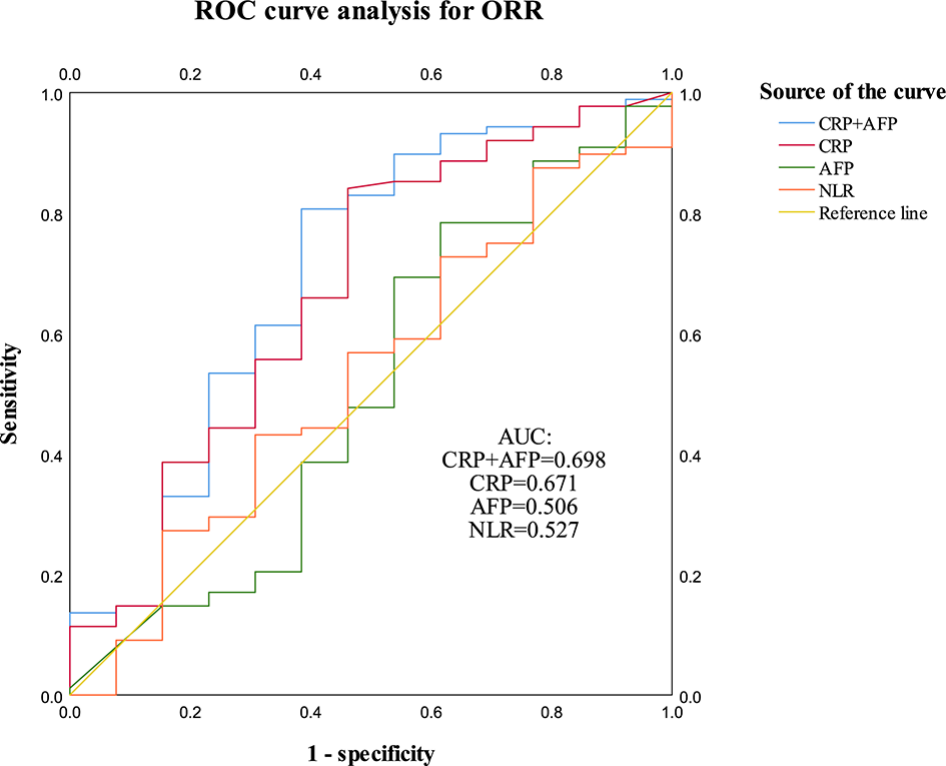
ROC curve analysis for CRP, AFP and NLR was performed to evaluate response (CR + PR) of patients to PD-1 inhibitors. CRP (AUC = 0.671, *P =* 0.047) and CRP + AFP (AUC = 0.698, *P =* 0.021) implied statistical associations with treatment response to the PD-1 inhibitors.

To identify the relationship between clinicopathological factors and the PFS of HCC patients treated with PD-1 inhibitors, univariate Cox proportional models were used, which identified elevated serum CRP, NLR and AFP levels as possible prognostic factors for the PFS of HCC patients. The multivariate Cox proportional analysis validated the finding that elevated serum CRP and AFP levels were significant and independently prognostic factors of PFS but not NLR. The other variables investigated did not show any prognostic value in predicting PFS (**Table 2**). With regard to overall survival, we also found that the elevated CRP level was an independently prognostic variable for OS (**Supplemental Table 1**). Because of the positive correlation between CRP levels and tumor size and NLR in baseline characteristics (**Table 1**), propensity score matching was applied to eliminate the confounding effect. CRP and AFP were still the independently prognostic variables after matching (**Supplemental Tables 2** **,** **3**). Moreover, ROC curve analysis further showed that CRP level had the highest AUC value for predicting the treatment response (CR + PR) (**Figure 2**).

**Table 2:** Univariate and multivariate Cox regression analyses of risk factors for progression-free survival.

	Univariate	*p* value	Multivariate	*p* value
	HR (95% CI)		HR (95% CI)	
**Age, y**				
≤60	1.0			
>60	0.88 (0.45–1.75)	0.720		
**Gender**				
Male	1.0			
Female	0.71 (0.39–1.28)	0.253		
**Aetiology**				
Viral hepatitis	1.0			
Other	1.97 (0.60–6.40)	0.262		
**Child-Pugh grade**				
A	1.0			
B	1.58 (0.74–3.34)	0.237		
**ECOG PS**				
0	1.0			
≥1	0.89 (0.52–1.52)	0.669		
**CRP level**				
≤20.9	1.0			
>20.9	2.09 (1.20–3.65)	0.009	1.83 (1.04–3.20)	0.036
**AFP, ng/ml**				
≤400	1.0			
>400	3.0 (1.68–5.35)	<0.001	2.81 (1.57–5.04)	0.001
**WBC count (10^9/L)**				
<10	1.0			
>10	0.98 (0.30–3.13)	0.996		
**Neutrophil-lymphocyte ratio (NLR)**				
<3	1.0			
≥3	1.97 (1.16–3.33)	0.012		
**Tumor size, cm**				
≤5	1.0			
>5	1.13 (0.67–1.90)	0.649		
**Tumor number**				
Single	1.0			
Multiple	1.31 (0.75–2.27)	0.342		
**Macrovascular invasion**				
−	1.0			
+	1.18 (0.70–1.98)	0.540		
**Extrahepatic metastasis**				
−	1.0			
+	1.43 (0.84–2.44)	0.184		

AFP, α- fetoprotein; CI, confidence interval; CRP, C-reactive protein; ECOG PS, Eastern Cooperative Oncology Group Performance Status; HR, hazard ratio; NLR, Neutrophil-lymphocyte ratio; PFS, progression-free survival.

**Figure 3:**
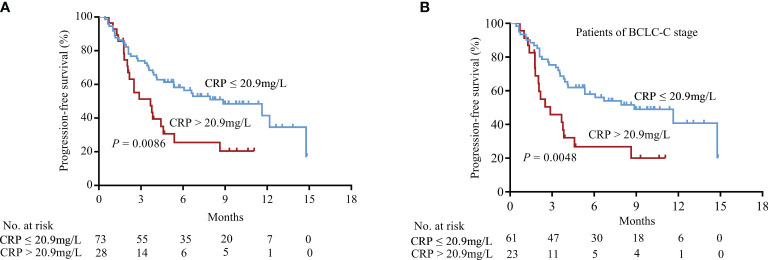
Association between CRP levels and PFS in HCC patients treated with PD-1 blockade. **(A)** Patients in the CRP-low group had more prolonged PFS than those in the CRP-high group (log-rank test). **(B)** In patients of BCLC-C stage, CRP levels also significantly discriminated patients with a poor prognosis.

Kaplan-Meier curves based on serum CRP levels were generated, as shown in **Figure 3**. Elevated CRP levels significantly correlated with a poor PFS (**Figure 3A**, *P* = 0.0086). Furthermore, CRP levels could further stratify patients with BCLC-C stage HCC by predicting their PFS (**Figure 3B**, *P* = 0.0048). Kaplan-Meier curve analyses for overall survival showed the similar results of PFS (**Supplemental Figure 1**, *P* = 0.0002).

### Relationship Between Tumor Response for PD-1 Inhibitors and CRP-AFP Model

**Figure 4:**
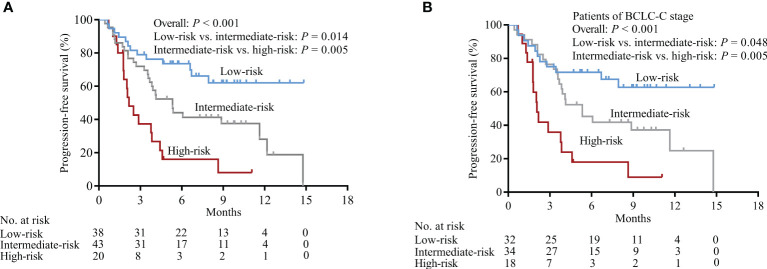
Association between the CRP-AFP model and PFS in HCC patients treated with PD-1 blockade. Patients with both, either, or none of elevated serum CRP and AFP levels were classified into high-, intermediate-, or low-risk subgroups. **(A)** All patients were stratified into markedly different risk subgroups by CRP-AFP model. **(B)** In patients with BCLC-C stage, the CRP-AFP model succeeded in stratifying them into low-, intermediate-, or high-risk subgroups.

**Figure 5:**
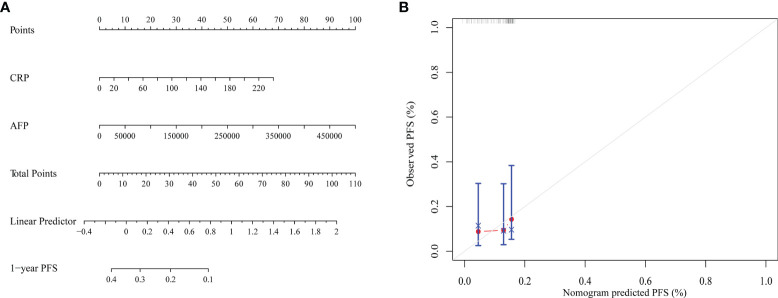
**(A)** Nomogram for predicting the PFS of HCC patients with CRP and AFP. **(B)** The calibration curve for predicting patient PFS at 1 year.

Due to the markedly heterogeneous prognosis of HCC patients treated with immunotherapies, an additional prognostic model based on serum CRP and AFP levels was constructed to more precisely predict the outcomes of patients. Patients that had both, either, or no elevated levels of serum CRP and AFP were classified into high-, intermediate-, or low-risk subgroups, respectively. The PFS results of HCC patients receiving PD-1 inhibitors were significantly different for the different risk subgroups (**Figure 4A**, *P* < 0.001). Patients with the BCLC-C stage were also stratified into different risk subgroups (**Figure 4B**, *P* < 0.001). The ROC curve analysis showed that CRP-AFP model presented the best performance in predicting the treatment response (CR + PR) of patients treated with PD-1 inhibitors (**Figure 2**). Nomogram further confirmed the predictive value of CRP and AFP for patients’ PFS (**Figure 5**).

The relationships between the CRP-AFP model and the response of a patient to PD-1 inhibitors were summarized in **Table 3**. Based on RECIST v1.1, the responses of the low-risk group included a complete response (CR) [2.6% (1/38)], partial response (PR) [10.5% (4/38)], stable disease (SD) [68.4% (26/38)], and progressive disease (PD) [18.4% (7/38)]. Among the patients in the intermediate-risk group, 0 patients were reported to have a CR, 11.6% (5/43) were reported to have PR, 53.5% (23/43) were reported to have SD, and 34.9% (15/43) were reported to have PD. Among the patients in the high-risk group, no patients were reported to have a CR, 5% (1/20) exhibited PR, 30% (6/20) were reported to have SD, and 65% (13/20) were reported to have PD. The overall response rates (ORR, CR + PR) were 13.2% (5/38) in the low-risk group, 11.6% (5/43) in the intermediate-risk group, and 5% (1/20) in the high-risk group (*P* = 0.625). The disease control rates (DCR, CR + PR + SD) were 81.6% (31/38) in the low-risk group, 65.1% (28/43) in the intermediate-risk group, and 35% (7/20) in the high-risk group (*P* = 0.002).

**Table 3:** The relationship between tumor response and occurrence of CRP-AFP model in patients treated with PD-1 inhibitors.

Treatment response	CRP-AFP model			p
	Low-riskgroup (n = 38)	Intermediate-risk group (n = 43)	High-risk group (n = 20)	
**CR**	1 (2.6)	0 (0.0)	0 (0.0)	
**PR**	4 (10.5)	5 (11.6)	1 (5.0)	
**SD**	26 (68.4)	23 (53.5)	6 (30.0)	
**PD**	7 (18.4)	15 (34.9)	13 (65.0)	
**ORR (CR+PR)**	5 (13.2)	5 (11.6)	1 (5.0)	0.625
**DCR (CR+PR+SD)**	31 (81.6)	28 (65.1)	7 (35.0)	0.002

Variables are expressed as number of patients (%).

AFP, alpha-fetoprotein; CR, complete response; CRP, C-reactive protein; DCR, disease control rate; ORR, overall response rate; PD, progressive disease; PR, partial response; SD, stable disease.

## Discussion

The use of immune checkpoint inhibitors, especially PD-1 inhibitors, have revolutionized the treatment landscape of patients with advanced HCC. However, due to the molecular heterogeneity of advanced HCC, even patients within the same subgroup have been reported to have significantly different outcomes to treatment. Prognostic indicators for the use of PD-1 inhibitors are urgently needed. Previous research suggests CRP may serve as a useful biomarker; however, its prognostic value in HCC patients treated with PD-1 inhibitors is still unclear. The novel results of this study showed a strong association between CRP levels and the response of HCC patients to PD-1 inhibitors. It is of particular interest that the CRP levels in combination with AFP levels, in patients prior to receiving treatment, could effectively determine risk stratification.

Previous reports of prognostic indicators of PD-1 inhibitors have proven to be disappointing. PD-L1 expression was proposed to be a prognostic factor in early studies investigating the safety and efficacy of PD-1 and PD-L1 inhibitors. However, analyses of PD-L1 with different assays using the same samples had discordant results (16). Another analysis of PD-L1 expression failed to discriminate between responders and non-responders (17). Tumor mutational burden (TMB) has been demonstrated to be a useful biomarker for immune checkpoint inhibitors (ICI) selection across some cancer types (18). However, data on the significance of TMB in HCC patients are scarce (19). Mismatch repair deficiency can be used to predict the response of solid tumors to PD-1 inhibitors, however, only 2-3% of HCC patients have been identified to have microsatellite instability-high tumors (20). Furthermore, the methods to investigate the biomarkers mentioned above are expensive, sometimes fail to predict therapy response, and have had contradictory results. Consequently, biomarkers with the following features are urgently needed: simple, widely applicable, cost-effective, and efficient.

As an acute inflammatory response biomarker, serum CRP has been recognized as an indicator of progression for several malignant tumors (21, 22). AFP levels > 400 μg/L have also been associated with decreased survival and are used as a biomarker to select HCC patients for treatment with the VEGFR2 inhibitor ramucirumab (23, 24). However, there are no previous studies that focused on the prognostic significance of pre-immunotherapy serum CRP levels in the response to PD-1, or the prognostic value of CRP combined with AFP levels. To address the gap, our multivariate COX analysis demonstrated that elevated serum CRP and AFP levels were significant and independent prognostic variables for a poor PFS. CRP levels were found to have the highest predictive value in determining the response (CR + PR) to the PD-1 inhibitors. We also demonstrated the discriminative power of the CRP-AFP prognostic model, which was strong enough to stratify different risk subgroups of patients [low-, intermediate-, and high-risk groups with significantly different DCR (81.6%, 65.1%, and 35%, respectively, *P* = 0.002)]. Our study also assessed the prognostic value of CRP levels with respect to BCLC-C stage patients, which showed that elevated CRP levels also significantly predicted a poor prognosis. Additionally, the CRP-AFP combined model could stratify BCLC-C stage patients into different risk subgroups. Compared with MSI-H, TMB, and PD-L1 expression, serum CRP and AFP levels could prospectively predict both the prognosis and response to PD-1 inhibitors prior to treatment. Furthermore, because these biomarkers are simple, cheap, reproducible, and objective, they may represent a prognostic indicator with more widespread clinical application value.

To the best of our knowledge, primary and acquired resistance to anti-PD1/PDL1 therapy might occur as a result of insufficient antigen immunogenicity, dysfunction of antigen presentation, irreversible T cell exhaustion, resistance of IFN-γ signaling, and immunosuppressive TME (25). The resistance to immune checkpoint blockade can result from disruptions in any of these key tumor characteristics, either by preventing a *de novo* antitumor immune response or by counteracting an ongoing antitumor response. CRP is an acute phase protein induced by IL-6, produced in the liver, and released into the circulation system, is involved in opsonization for phagocytosis, the complement system activation, and direct-binding with Fc receptor. CRP plays extensive roles in innate and adaptive immune systems (26, 27). Zhang et al. (28) had demonstrated that elevated CRP levels *in vitro* inhibited Th1 differentiation and augmented Th2 differentiation of naive CD4(+) T cells. In the experimental mouse model for autoimmune encephalomyelitis, elevated CRP levels inhibited both the Th1 cell response and disease severity. Jimenez et al. (29) found that elevated CRP levels could reduce the yield of CD11c^+^ bone marrow-derived dendritic cells (BMDCs), prevented their full expression of MHC II and the co-stimulatory molecules CD86 and CD40, and decreased the ability of BMDCs to stimulate antigen-driven proliferation of T cells *in vitro*. Data from CheckMate 064, 066 and 067 also found that elevated levels of IL-6 and CRP played both an indirect and a direct suppressive role, accounting for the poor outcomes of melanoma patients receiving single agent and combination checkpoint inhibition. This may be explained by CRP binding to T cells and suppressing their function in a dose dependent manner at the initial stages of T-cell activation (12, 30). Additionally, IL-6, a chronic inflammatory protein that induces CRP expression, was also reported to have a potential immune suppressive function and promote tumor progression by inducing tumor cell expression of STAT3 and its downstream target genes. Elevated levels of IL-6 are also associated with the formation of desmoplastic tumor stroma and can also stimulate the generation of MDSC cells in coordination with TGF-β. Thus, elevated IL-6 and CRP may both play important roles in the poor outcomes observed for HCC patients treated with PD-1 inhibitors. Unfortunately, our study did not detect serum IL-6 levels.

Several studies reported a possible negative influence of systemic antibiotics on HCC, and other tumor types, when given within 30 days prior or after the initiation of ICIs (31–33). Primary resistance to ICIs can be attributed to abnormal gut microbiome composition. However, in our study, only several patients had received one-time preventative intravenous antibiotics due to HAIC therapy. No patient reported potential factors that would interfere with CRP levels, such as acute or chronic inflammation, infection, or trauma. Thus, no patient with elevated CRP levels received systemic antibiotics to deal with one of the aforementioned situations, which precluded the possible negative effect of systemic antibiotics mediated by elevated CRP.

There are a few limitations in this study. CRP is a non-specific inflammatory factor and a sensitive acute-phase protein, which could be affected by many factors, such as acute and chronic inflammation, undocumented infection, and trauma. Thus, serum CRP levels should be detected during the stable phase of disease to avoid potential interference of other factors. Additionally, this is a retrospective study, and still necessitates prospective studies to verify its conclusions.

In conclusion, the results of this study demonstrated the prognostic value of pretreatment serum CRP levels for advanced HCC patients and treatment with PD-1 inhibitors. Additionally, the CRP-AFP prognostic model could differentiate the HCC patients with the highest potential to benefit from PD-1 inhibitors. This would help avoid unnecessary therapy, efficiently saving time and reducing overall medical expenses as patients would receive more precise and appropriate treatments.

## Data Availability Statement

The raw data in this paper has been successfully uploaded and locked onto Research Data Deposit (www.researchdata.org.cn) with a RDD number of 2109280001.

## Ethics Statement

The studies involving human participants were reviewed and approved by the ethics and research committee of Sun Yat-sen University Cancer Center. The patients/participants provided their written informed consent to participate in this study.

## Author Contributions

MYC and XC contributed to conception and design of the study. YYZ, LL and ZH completed the work of follow-up, carried out the initial analysis, and prepared the first draft of manuscript. MYC and XC critically reviewed and revised the manuscript. ZSX, ZCX, RCN, WL, WC, JZ, YY, JX, YCZ, XZ, TZ, XXC, and PL conducted the research and collected the data. All authors contributed to manuscript revision, read, and approved the submitted version.

## Funding

This study was supported by grants from National Key R&D Program of China, grant/award number: 2017YFC1309000; National Natural Science Foundation of China, grant/award numbers: 81672407, 81872001, 82172646.

## Conflict of Interest

The authors declare that the research was conducted in the absence of any commercial or financial relationships that could be construed as a potential conflict of interest.

## Publisher’s Note

All claims expressed in this article are solely those of the authors and do not necessarily represent those of their affiliated organizations, or those of the publisher, the editors and the reviewers. Any product that may be evaluated in this article, or claim that may be made by its manufacturer, is not guaranteed or endorsed by the publisher.

## Supplementary Material

Supplementary Table 1 Univariate and multivariate Cox regression analyses of risk factors for OS. AFP, α- fetoprotein; CI, confidence interval; CRP, C-reactive protein; ECOG PS, Eastern Cooperative Oncology Group Performance Status; HR, hazard ratio; NLR, Neutrophil-lymphocyte ratio; OS, overall survival; PFS, progression-free survival.

Supplementary Table 2 Baseline characteristics of AFP, NLR and tumor size in CRP subgroups after Propensity Score Matching. AFP, α- fetoprotein; CRP, C-reactive protein; NLR, Neutrophil-lymphocyte ratio.

Supplementary Table 3 Univariate and multivariate Cox regression analyses of risk factors for progression-free survival after Propensity Score Matching in the context of 1:2 matching. AFP, α-fetoprotein; CI, confidence interval; CRP, C-reactive protein; HR, hazard ratio; NLR, Neutrophil-lymphocyte ratio.

Supplementary Figure 1 Association between CRP & AFP levels and OS in HCC patients treated with PD-1 blockade. **(A)** Patients in the CRP-low group had more prolonged OS than those in the CRP-high group (log-rank test, *P =* 0.0002). **(B)** Patients in the AFP-low group presented more prolonged OS (*P =* 0.0002). **(C)** Patients of high-risk group showed a significantly pooper OS than the other two groups.

Supplementary Figure 2 ROC curve analysis for CRP, AFP, NLR, WBC, and tumor size was performed to evaluate tumor progression of patients receiving anti-PD-1 therapy.

## Abbreviations

AFP, α-fetoprotein; BCLC stage, Barcelona Clinic Liver Cancer stage; CI, confidence interval; CRP, C-reactive protein; ECOG PS, Eastern Cooperative Oncology Group performance status; HAIC, hepatic arterial infusion of chemotherapy; HCC, hepatocellular carcinoma; HR, hazard ratio; NLR, neutrophil-lymphocyte ratio; PFS, progression-free survival; TACE, transcatheter arterial chemoembolization.
