# Supplementary material for: C-Reactive Protein Levels Predict Responses to PD-1 Inhibitors in Hepatocellular Carcinoma Patients
Source: Front Immunol. 2022 Feb 4;13:808101. doi: 10.3389/fimmu.2022.808101 (PMC8854259; doi:10.3389/fimmu.2022.808101)
Supplement: Supplementary Table 1 — Univariate and multivariate Cox regression analyses of risk factors for OS. AFP, α- fetoprotein; CI, confidence interval; CRP, C-reactive protein; ECOG PS, Eastern Cooperative Oncology Group Performance Status; HR, hazard ratio; NLR, Neutrophil-lymphocyte ratio; OS, overall survival; PFS, progression-free survival. [file Table_1.docx]

| **Supplemental Table 1. Univariate and multivariate Cox regression analyses of risk factors for OS.** | | | | |
| --- | --- | --- | --- | --- |
|  | **Univariate** |  | **Multivariate** |  |
|  | **HR (95% CI)** | ***p* value** | **HR (95% CI)** | ***p* value** |
| **Age, y** |  |  |  |  |
| ≤60 | 1.0 |  |  |  |
| >60 | 1.36 (0.62-3.03) | 0.445 |  |  |
| **Gender** |  |  |  |  |
| Male | 1.0 |  |  |  |
| Female | 1.71 (0.65-4.50) | 0.278 |  |  |
| **Child-Pugh grade** |  |  |  |  |
| A | 1.0 |  |  |  |
| B | 2.04 (0.84-4.96) | 0.115 |  |  |
| **ECOG PS** |  |  |  |  |
| 0 | 1.0 |  |  |  |
| ≥1 | 1.25 (0.62-2.53) | 0.537 |  |  |
| **CRP level** |  |  |  |  |
| ≤20.9 | 1.0 |  |  |  |
| >20.9 | 3.48 (1.75-6.93) | 0.000 | 3.48 (1.75-6.93) | 0.000 |
| **AFP, ng/ml** |  |  |  |  |
| ≤400 | 1.0 |  |  |  |
| >400 | 2.06 (1.00-4.24) | 0.49 |  |  |
| **WBC count (10^9/L)** |  |  |  |  |
| <10 | 1.0 |  |  |  |
| >10 | 3.73 (1.53-9.06) | 0.004 |  |  |
| **NLR** |  |  |  |  |
| <3 | 1.0 |  |  |  |
| ≥3 | 2.31 (1.17-4.59) | 0.016 |  |  |
| **Tumor size, cm** |  |  |  |  |
| ≤5 | 1.0 |  |  |  |
| >5 | 1.17 (0.54-2.53) | 0.687 |  |  |
| **Macrovascular invasion** |  |  |  |  |
| - | 1.0 |  |  |  |
| + | 1.02 (0.52-2.00) | 0.959 |  |  |
| **Extrahepatic metastasis** |  |  |  |  |
| - | 1.0 |  |  |  |
| + | 1.74 (0.83-3.63) | 0. 142 |  |  |

Abbreviations: AFP, α- fetoprotein; CI, confidence interval; CRP, C-reactive protein; ECOG PS, Eastern Cooperative Oncology Group Performance Status; HR, hazard ratio; NLR, Neutrophil-lymphocyte ratio; OS, overall survival; PFS, progression-free survival.

| **Supplemental Table 2. Baseline characteristics of AFP, NLR and tumor size in CRP subgroups after Propensity Score Matching** | | | | |
| --- | --- | --- | --- | --- |
|  | Total  (n = 54) | CRP > 20.9 (n = 22) | CRP ≤ 20.9 (n = 32) | *P value* |
| **Tumor size (cm)** |  |  |  | 0.433 |
| ≤ 5 | 18 (33.3) | 6 (27.3) | 12 (37.5) |  |
| > 5 | 36 (66.6) | 16 (72.7) | 20 (62.5) |  |
| **Neutrophil-lymphocyte ratio (NLR)** |  |  |  | 0.587 |
| <3 | 22 (40.7) | 8 (36.4) | 14 (43.8) |  |
| ≥3 | 32 (59.3) | 14 (63.6) | 18 (56.3) |  |
| **AFP level (ng/l)** |  |  |  | 0.753 |
| ≤ 400 | 16 (29.6) | 6 (27.3) | 10 (31.3) |  |
| > 400 | 38 (70.4) | 16 (72.7) | 22 (68.8) |  |

AFP, α- fetoprotein; CRP, C-reactive protein; NLR, Neutrophil-lymphocyte ratio.

| **Supplemental Table 3. Univariate and multivariate Cox regression analyses of risk factors for progression-free survival after Propensity Score Matching in the context of 1:2 matching.** | | | | |
| --- | --- | --- | --- | --- |
|  | **Univariate** |  | **Multivariate** |  |
|  | **HR (95% CI)** | ***p* value** | **HR (95% CI)** | ***p* value** |
| **Tumor size, cm** |  |  |  |  |
| ≤5 | 1.0 |  |  |  |
| >5 | 1.01 (0.49-2.07) | 0.987 |  |  |
| **NLR** |  |  |  |  |
| <3 | 1.0 |  |  |  |
| ≥3 | 1.49 (0.72-3.06) | 0.279 |  |  |
| **CRP level** |  |  |  |  |
| ≤20.9 | 1.0 |  |  |  |
| >20.9 | 2.16 (1.09-4.25) | 0.027 | 2.33 (1.17-4.62) | 0.015 |
| **AFP, ng/ml** |  |  |  |  |
| ≤400 | 1.0 |  |  |  |
| >400 | 3.41 (1.40-8.31) | 0.007 | 3.63 (1.48-8.87) | 0.005 |

AFP, α-fetoprotein; CI, confidence interval; CRP, C-reactive protein; HR, hazard ratio; NLR, Neutrophil-lymphocyte ratio.
