# Supplementary figures and images for: C-Reactive Protein Levels Predict Responses to PD-1 Inhibitors in Hepatocellular Carcinoma Patients
Source: Front Immunol. 2022 Feb 4;13:808101. doi: 10.3389/fimmu.2022.808101 (PMC8854259; doi:10.3389/fimmu.2022.808101)

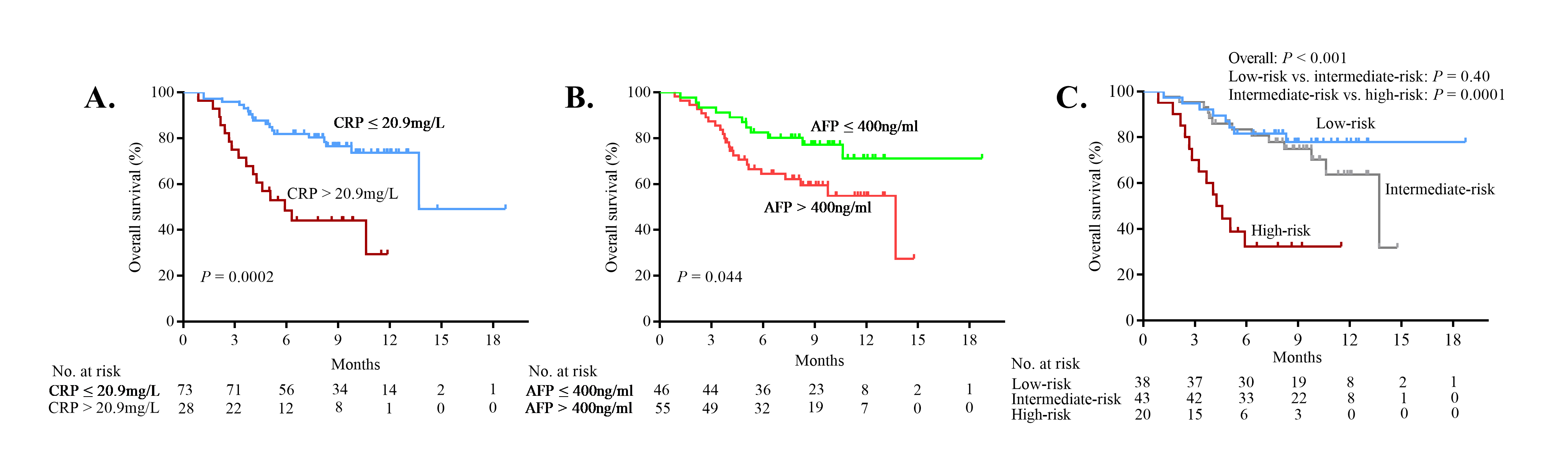

Supplement: Supplementary Figure 1 — Association between CRP & AFP levels and OS in HCC patients treated with PD-1 blockade. (A) Patients in the CRP-low group had more prolonged OS than those in the CRP-high group (log-rank test, P = 0.0002). (B) Patients in the AFP-low group presented more prolonged OS (P = 0.0002). (C) Patients of high-risk group showed a significantly pooper OS than the other two groups. [file Image_1.tif]

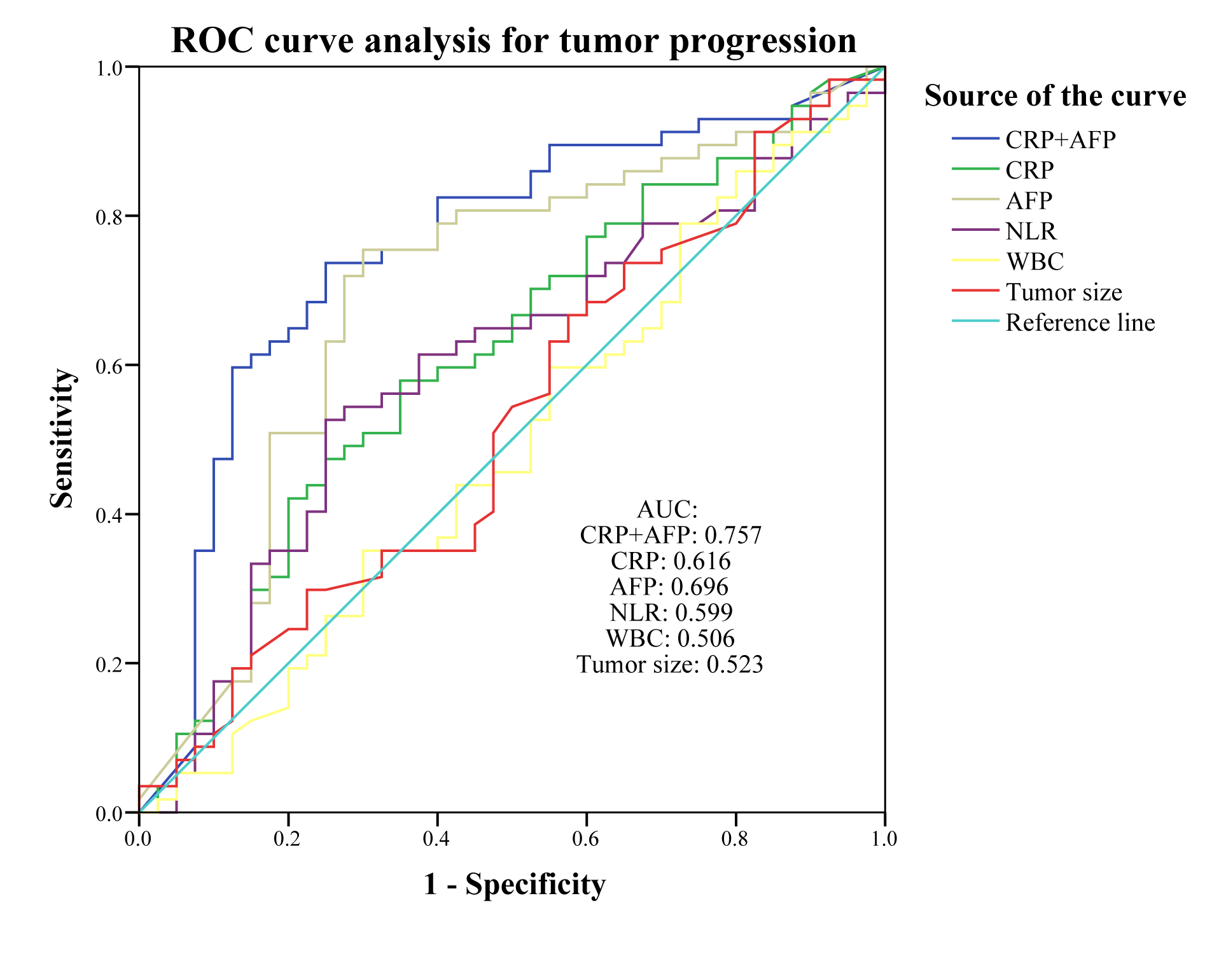

Supplement: Supplementary Figure 2 — ROC curve analysis for CRP, AFP, NLR, WBC, and tumor size was performed to evaluate tumor progression of patients receiving anti-PD-1 therapy. [file Image_2.tif]
